# Titania nanotubes prepared by rapid breakdown anodization for photocatalytic decolorization of organic dyes under UV and natural solar light

**DOI:** 10.1186/s11671-018-2591-5

**Published:** 2018-06-14

**Authors:** Saima Ali, Henrika Granbohm, Jouko Lahtinen, Simo-Pekka Hannula

**Affiliations:** 1 Department of Chemistry and Materials Science, Aalto University School of Chemical Engineering, P.O. Box 16100, FI-00076 Espoo, Finland; 2 Department of Applied Physics, School of Science, Aalto University, P.O. Box 15100, FI 00076 Espoo, Finland

**Keywords:** Photocatalysis, Titania nanotubes, Bandgap, Methyl orange, Rhodamine B

## Abstract

Titania nanotube (TNT) powder was prepared by rapid breakdown anodization (RBA) in a perchloric acid electrolyte. The photocatalytic efficiency of the as-prepared and powders annealed at temperatures between 250 and 550 °C was tested under UV and natural sunlight irradiation by decolorization of both anionic and cationic organic dyes, i.e., methyl orange (MO) and rhodamine B (RhB), as model pollutants. The tubular structure of the nanotubes was retained up to 250 °C, while at 350 °C and above, the nanotubes transformed into nanorods and nanoparticles. Depending on the annealing temperature, the TNTs consist of anatase, mixed anatase/brookite, or anatase/rutile phases. The bandgap of the as-prepared nanotubes is 3.04 eV, and it shifts towards the visible light region upon annealing. The X-ray photoelectron spectroscopy (XPS) results show the presence of titania and impurities including chlorine on the surface of the TNTs. The atomic ratio of Ti/O remains unchanged for the annealed TNTs, but the concentration of chlorine decreases with temperature. The photoluminescence (PL) indicate high electron-hole recombination for the as-prepared TNTs, probably due to the residual impurities, low crystallinity, and vacancies in the structure, while the highest photocurrent was observed for the TNT sample annealed at 450 °C. The TNTs induce a small degradation of the dyes under UV light; however, contrary to previous reports, complete decolorization of dyes is observed under sunlight. All TNT samples showed higher decolorization rates under sunlight irradiation than under UV light. The highest reaction rate for the TNT samples was obtained for the as-prepared TNT powder sample under sunlight using RhB (*κ*_1_ = 1.29 h^−1^). This is attributed to the bandgap, specific surface area and the crystal structure of the nanotubes. The as-prepared TNTs performed most efficiently for decolorization of RhB and outperformed the reference anatase powder under sunlight irradiation. This could be attributed to the abundance of reactive sites, higher specific surface area, and degradation mechanism of RhB. These RBA TNT photocatalyst powders demonstrate a more efficient use of the sunlight spectrum, making them viable for environmental remediation.

**Electronic supplementary material:**

The online version of this article (10.1186/s11671-018-2591-5) contains supplementary material, which is available to authorized users.

## Background

One-dimensional titania nanotubes (TNT) have attracted much attention in the past decade. They have been studied for a number of prospective applications, due to their promising electrical and optical properties and high specific surface area [1]. The most promising research on TNT has been done for gas sensing, photocatalysis, dye-sensitized solar cells, electrochromic devices, and biomedical applications [2]. TNTs can be synthesized by various methods including electrochemical anodization, hydrothermal processing, chemical processing, template-assisted, and sol-gel methods [1–4]. In template-free methods, Zwilling et al. [5] introduced the preparation of TNT arrays by electrochemical anodization of titanium foil in 1999. The research on anodization of titanium has continued to control the morphology and dimensions of TNT arrays by altering parameters such as electrolytes, electrolyte pH, temperature, applied potential, current density, and anodization time [6, 7]. However, making larger batches of TNT arrays, to further peel off the arrays from the Ti-substrate to obtain powders [8], is time-consuming. Therefore, a faster method called rapid breakdown anodization (RBA) was developed, using chloride and perchlorate ions in an electrolyte [7, 9]. In RBA, metallic titanium is transformed into metal-oxide nanotubes within seconds after the application of voltage. When the voltage is applied, a thin layer of oxide is grown on the native oxide of the metal surface, which is quickly attacked by halide ions to form the localized pits on the metal surface [10]. The pitting process is affected by the applied voltage, temperature, and halide concentration [10]. After pitting, the oxide layer forms within the pits by the inward migration of O^2−^ ions from the electrolyte to the metal surface [11]. The oxidized metal ions (Ti^4+^) migrate outwards, and the formed TiO_2_ layer is etched by the chloride ions to form water-soluble [TiCl_6_]^2−^ ions [9]. Nanotubes are formed when the equilibrium is maintained in the oxide growth and chemical etching of oxide [9, 11]. The bundles of nanotubes grow around the pits in a random direction within few seconds. After some time, the nanotube bundles fall into the electrolyte due to high stress between the metal and the oxide nanotubes [9]. The nanotube powder is then collected from the electrolyte [11]. TNT powders prepared by RBA have been studied for a number of potential applications, such as DSSC [12, 13], hydrogen generation [14–19], photocatalytic degradation of pollutants under UV light irradiation [20–22], and biomedical implants [23, 24].

Titanium dioxide has been utilized for the removal of organic pollutants from the water stream, as it is non-toxic, thermally stable, inexpensive and easily available material [25]. TNTs have been studied for the photocatalytic degradation of organic pollutants in the water using different dyes as model pollutants [25–27]. TNTs have a high specific surface area and are either amorphous or crystalline structure depending on the synthesis process. The crystal structure can be modified by annealing with simultaneously affecting the morphology, bandgap, composition, and specific surface area of the nanotubes [3, 11]. The bandgap of the TNTs is reported to be in the range of 3.0–3.2 eV [2] depending on the crystal structure of the nanotubes. The crystal structure of the nanotubes is dependent on the synthesis conditions and can be modified to the three polymorphs of TiO_2_, i.e., anatase, rutile, and brookite. Anatase has an indirect bandgap and possesses a longer electron-hole lifetime compared to the direct bandgap of rutile and brookite phase [28]. Nanotubes are expected to have improved photocatalytic efficiency due to their tubular morphology, larger specific surface area, and wider bandgap [29] under UV light irradiation. Consequently, a number of studies report the efficacy of TNT arrays for the degradation of organic dyes by UV light irradiation [25–27]. As UV light is only a small portion of the sunlight spectrum, a more efficient utilization of solar energy for environmental remediation also requires the use of the visible light range. However, TNTs are inefficient photocatalysts under visible light irradiation [29]. To improve the photocatalytic efficiency of TNTs in the visible light range, their electronic properties are usually modified by anionic (non-metal ions) or cationic (metal ions) doping [27, 30–32].

In the present study, undoped TNT powders were prepared by RBA and subjected to annealing from 250 to 550 °C for 3 h in air. The resulting TNT powders are examined as photocatalysts under UV and natural sunlight irradiation by determining their efficiency by decolorization of Rhodamine B (RhB) and methyl orange (MO) dyes. The dyes are stable with unique colors and have been used in the paper, textile, cosmetics, and photographic industries [20]. A number of photocatalytic studies have been made on TNT arrays [32]; however, only a few reports have been presented for the photocatalytic degradation of organic pollutants by TNT powders prepared by RBA [20, 21, 33]. However, to the best of our knowledge, no dye degradation studies have been made under natural sunlight for TNTs prepared by RBA. In this study, we found that undoped TNTs prepared by RBA are more efficient under natural sunlight than under UV light, and complete decolorization of organic dyes is obtained under natural solar light irradiation. This suggests efficient utilization of the solar spectrum for environmental remediation, such as industrial wastewater purification.

## Methods/Experimental

### Preparation of the TNTs

TNT powder was prepared by using 0.1 M HClO_4_ electrolyte (Sigma-Aldrich, 70%) and an applied voltage of 20 V as described previously [11]. The as-prepared and annealed powders (250–550 °C, 3 h) were characterized to explore the morphology, composition, crystal structure, and specific surface area of the nanotubes.

### Characterization methods

The morphology was examined by transmission electron microscopy (TEM; Tecnai F-20G2 FEG S-twin GIF) at the operational voltage of 200 kV. X-ray diffraction (XRD) characterization was done to study the crystal structure of the TNTs using a PANalytical X’pert Pro diffractometer, and the measurements were made in two theta range of 20°–110° at an operating voltage of 40 kV and current of 40 mA, by using Co-Kα radiation having a wavelength of 0.179 nm. Raman spectroscopy was obtained using a Labram HR Raman Spectrometer by Horiba Jobin-Yvon equipped with argon laser excitement of 514 nm at 50 mW. The measurement was carried out on the TNT powders with × 50 objective (Olympus BX41). U*V*/Vis/NIR spectroscopy was used to obtain the absorption spectra for the calculation of the bandgap energies. Diffuse reflectance spectroscopy (DRS) measurements were performed using an Agilent Cary 5000 equipment with an integrating sphere. The measurement was carried out in the range of 200–800 nm and a calibrated sample of Spectralon was used for the baseline correction. Fourier transform infrared spectroscopy (FTIR) was carried out in attenuated total reflection (ATR) mode in the spectral range of 525–4000 cm^−1^, with a resolution of 4 cm^−1^, using a Nicolet 380 FTIR. X-ray photoelectron spectroscopy (XPS) was used to analyze the surface chemical composition. The measurements were made using Kratos Analytical AXIS Ultra system, equipped with a monochromatic Al K_α_ (1486.6 eV) X-ray source, and C 1s (284.8 eV) was used as the binding energy reference. Photoluminescence spectroscopy (PL) of the TNT powders was performed using a Perkin Elmer LS 50B Luminescence spectrometer equipped with a 20 W Xenon lamp at an excitation of 330 nm.

For photocurrent characterization, electrodes were prepared by deposition of TNT film on fluorine-doped tin oxide (FTO) glass. The films were prepared by drop casting using a suspension of the TNT powders in ethanol on the FTO glass followed by drying for 20 min in an oven at 70 °C in the air. Few drops of Nafion (Sigma-Aldrich; 5 wt.% in lower aliphatic alcohols and water) were added on the film, and the sample was dried again in an oven for 20 min at 70 °C in air. The photocurrent characterization was done at a voltage of 500 mV by using Jaissle IMP83 PC-T-BC potentiostat. The measurements were performed using a three-electrode setup using an Ag/AgCl reference electrode, Pt as a counter electrode and the TNT film deposited on FTO glass as a working electrode. 0.1M Na_2_SO_4_ (Sigma-Aldrich; ≥ 99.0%) was used as an electrolyte. The samples were irradiated by an Oriel 6365 150 W Xe-lamp in the range of 250–600 nm, where the incident wavelength was chosen using an Oriel Cornerstone 130 1/8 m monochromator. Electrochemical impedance spectroscopy (EIS) was carried out using a Gamry Referece 600+ potentiostat (Gamry Instruments). A three-electrode setup with an Ag/AgCl reference electrode (+ 0.199 V vs RHE, radiometer analytical), TNT working electrode, and a Pt wire counter electrode was used in the measurements. EIS was used to study electron transfer with the outer sphere redox probe Ru(NH_3_)_6_^2 +/3 +^ (5 mM in 1 M KCl). EIS was performed from 200 kHz to 100 mHz using an AC signal of 15 mV. The formal potential as determined with cyclic voltammetry was used as DC potential in the measurements. All measurements were conducted at room temperature.

### Photocatalysis of TNTs

**Fig. 1:**
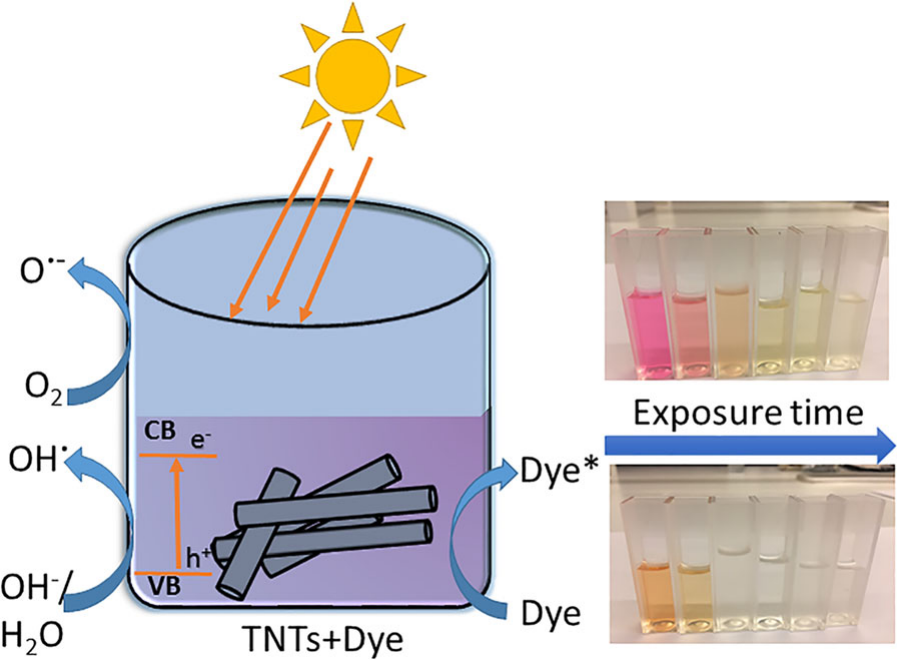
Schematic illustration of decolorization of organic dyes under natural solar light irradiation by undoped TNTs

The photocatalytic efficiency of the TNT powders was investigated by decolorization of aqueous solutions of anionic methyl orange (MO, Fluka, Reag. Ph. Eur) and cationic rhodamine B (RhB, Sigma, ~ 95%) dyes as model contaminants under UV and sunlight irradiation. The schematic illustration of the photocatalytic decolorization of the MO and RhB dyes by using TNTs as catalysts is shown in Fig. 1. The UV experiments were carried out in an in-house built reactor equipped with two Philips PL-S 11W/10/2P UV lamps. The UV lamps are in the wavelength range of 350–400 nm and intensity of 1 mW cm^−2^. Suspensions for the photocatalytic experiments were prepared by adding 100 mg TNT powder into 100 ml of 10 mg L^−1^ MO or RhB aqueous solutions. To check the adsorption capability of the catalysts, the dispersions were left under dark conditions for 3 h. The dispersions were then irradiated by UV or natural sunlight for 3 h to test the photocatalytic activity. Anatase TiO_2_ powder (Sigma-Aldrich, 99.8%) was used as reference material. Blank tests were also performed to measure the photolysis of MO and RhB under both UV and sunlight. The initial concentration (C_0_) of the suspensions were measured prior to irradiation at *t* = 0, i.e., after 3 h of dark adsorption. The concentration (C) of the samples was measured at given time intervals by separating the catalyst from the dye solutions by utilization of 0.45 μm Nylon syringe filters and centrifugation when necessary. The change in concentration (C/C_0_) is proportional to the change in absorbance (A/A_0_), where *A*_0_ is the initial absorbance at *t* = 0. The change in MO (*λ*_max_ = 465 nm) and RhB (*λ*_max_ = 554 nm) concentration is studied by recording the absorbance with a Hitachi U-5100 U*V*/Vis spectrophotometer. The minimum detection limit is 1.0 mg L^−1^ for MO and 0.1 mg L^−1^ for RhB. The natural sunlight experiments were carried out in Espoo, Finland (60°11′01.3″ N 24°49′32.2″ E) on completely sunny days around midday during June–July 2017.

## Results and discussion

### Morphology and structure

**Fig. 2:**
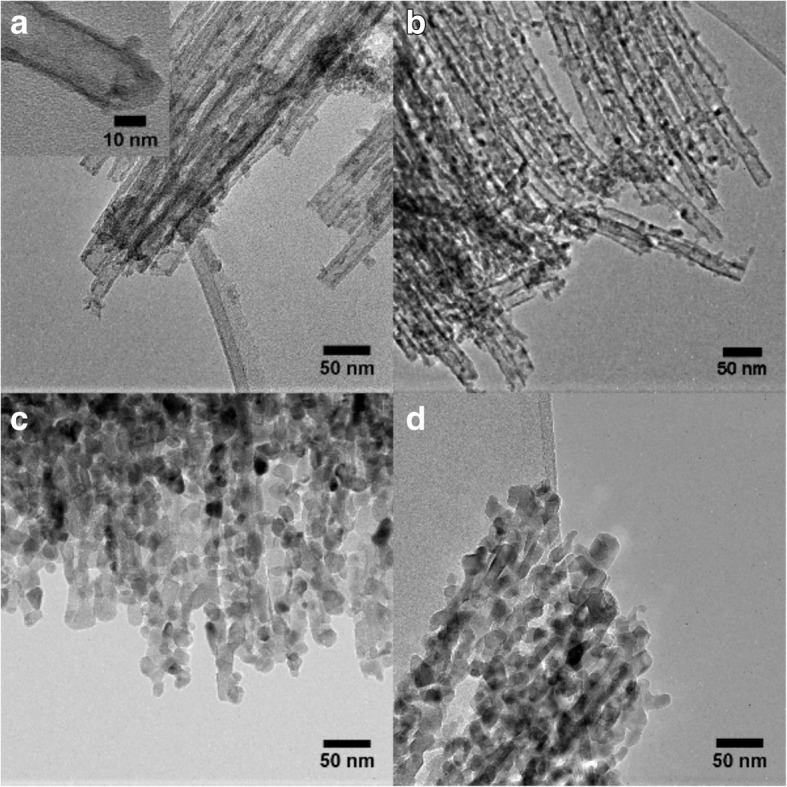
TEM images of TNTs. **a** As-prepared TNTs with one end open and other closed as shown in inset (figure inset scale bar = 10 nm), TNTs annealed at **b** 250 °C, **c** 350 °C (nanorod transformation), and **d** 450 °C

The single-walled TNT bundles are obtained within seconds after the application of the voltage in the RBA process. Nanotubes with an open top and closed bottom end, the pore size of 11–18 nm, and bundle length in the range of 18–35 μm are formed [11]. When the nanotubes are annealed at 250, 350, 450, and 550 °C for 3 h, changes in their morphology and crystal structure occur. These changes are discussed in details in ref. [11]. In brief, they can be summarized as follows: when annealed the nanotubes preserve their morphology up to 250 °C. At higher temperatures, the nanotubes transform to nanorods and nanoparticles as shown in Fig. 2a–d. This transformation results in the apparent decrease in the specific surface area during annealing at 350 °C and at higher temperatures, as listed in Table 2. The nanorod morphology obtained at higher temperatures, i.e., 350–550 °C is also shown in Additional file 1: Figure S1a–c.

**Fig. 3:**
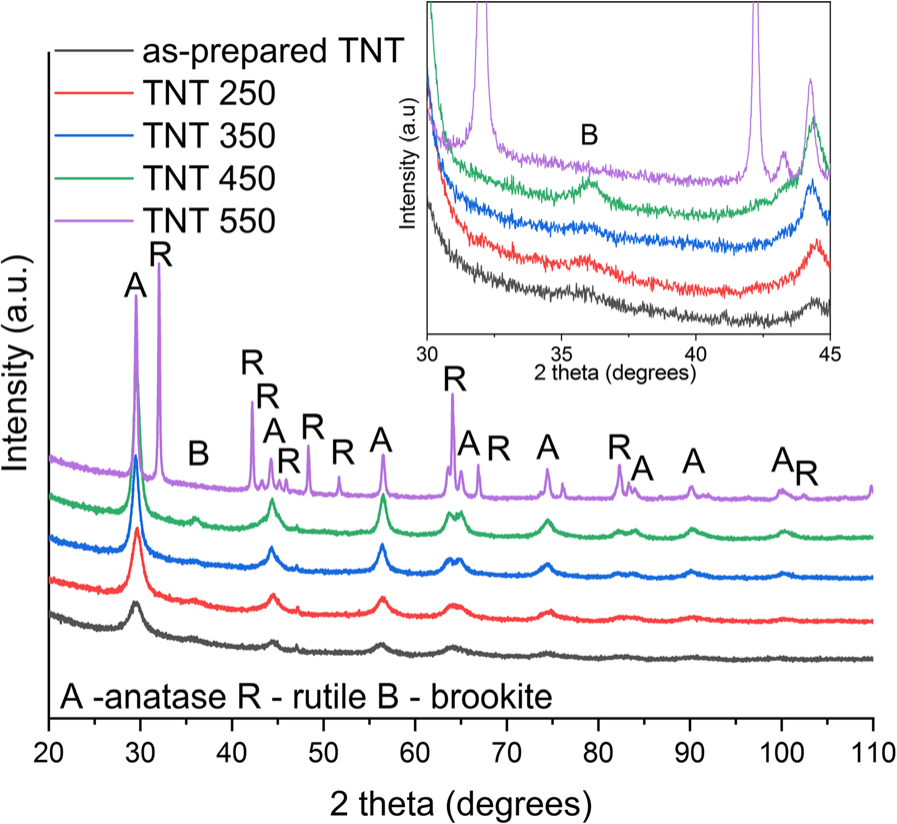
XRD of as-prepared and annealed TNTs by using Co K-alpha radiation

Figure 3 shows the XRD data of the as-prepared and annealed TNTs. The as-prepared TNTs are crystalline and display diffraction peaks from anatase phase. These results correspond well with the previous findings [11, 18, 34]. The anatase phase is also found for samples annealed at 250, 350, and 450 °C. In addition, TNT 450 and TNT 250 also show peaks from brookite phase, as shown in the inset of Fig. 3. The brookite peak disappears when the sample is annealed at 550 °C. TNT 550 displays peaks from anatase and rutile phase, similar to previous findings [11]. It should be noted that no brookite peaks were found after annealing in a previous study [34], but Preethi et al. [17] reported a brookite/anatase phase mixture for their as-prepared TNTs. The presence of brookite phase along with anatase in TNT 450 was also confirmed by the Raman spectra shown in Additional file 1: Figure S2. Raman data shows the presence of anatase phase in as-prepared TNT, TNT 250, and TNT 350, similar to previous findings [11]. TNT 550 shows the peaks from rutile phase, which supports the XRD results (Additional file 1: Figure S2). The weight percentages of each polymorph were approximated from the empirical equation proposed by Zhang and Banfield [35]. The proposed formulas are shown in Eq. 1a–c below [16, 35, 36].

1a $$ {W}_A=\frac{K_A{A}_A}{K_A{A}_A+{A}_R+{K}_B{A}_B} $$

1b $$ {W}_R=\frac{A_R}{K_A{A}_A+{A}_R+{K}_B{A}_B} $$

1c $$ {W}_B=\frac{K_B{A}_B}{K_A{A}_A+{A}_R+{K}_B{A}_B}, $$

where *W*_*A*_, *W*_*B*,_ and *W*_*R*_ represent the weight fractions of anatase, brookite, and rutile. *A*_*A*_ is the intensity of the (101) peak from anatase phase, *A*_*B*_ represents the intensity of the brookite (121), *A*_*R*_ represents the (110) peak intensity from the rutile phase, and *K*_*A*_ and *K*_*B*_ are the correction coefficients (*K*_*A*_ = 0.886 and *K*_*B*_ = 2.721) [35, 36]. The results suggest 64% of anatase phase and 36% of brookite phase for TNT 450 and 34% of anatase and 66% of rutile phase for TNT 550.

**Fig. 4:**
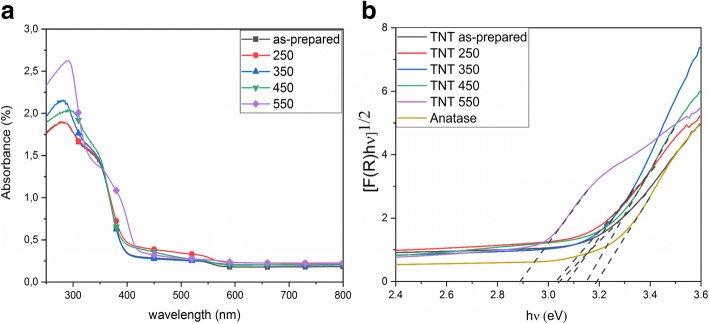
U*V*/Vis diffuse reflectance **a** absorbance spectra of as-prepared and annealed TNT **b** Kubelka-Munk function for bandgap estimation

The diffuse reflectance absorbance spectra of the TNT samples are shown in Fig. 4a. The diffuse reflectance spectra are used for the calculation of bandgap energy by applying Kubelka-Munk method (Fig. 4b). The bandgap of the as-prepared nanotubes is approximately 3.04 eV (Table 2). Similar bandgap sizes are found for the nanotubes annealed till 350 °C. TNT 450 and TNT 550 have a bandgap energy of 3.14 and 2.88 eV. A similar redshift towards visible light has also been observed for titanate nanotubes upon annealing [3]. This shift towards the visible light range is attributed to the change in the crystal structure of the nanotubes upon annealing, as rutile phase was observed at 550 °C in addition to anatase phase [11]. The earlier reported value for the bandgap of the rutile is 3.00 eV [2], and the experimental findings for the bandgap of brookite phase are in the range of 3.1–3.4 eV [37], which agrees well with our results. The bandgap of the as-prepared TNTs and the narrowing of the bandgap energies upon annealing are also in good agreement with previously published results [9, 34]. The bandgap energy of the reference anatase powder is 3.18 eV, which corresponds well to that of bulk anatase (3.23 eV) [37].

**Fig. 5:**
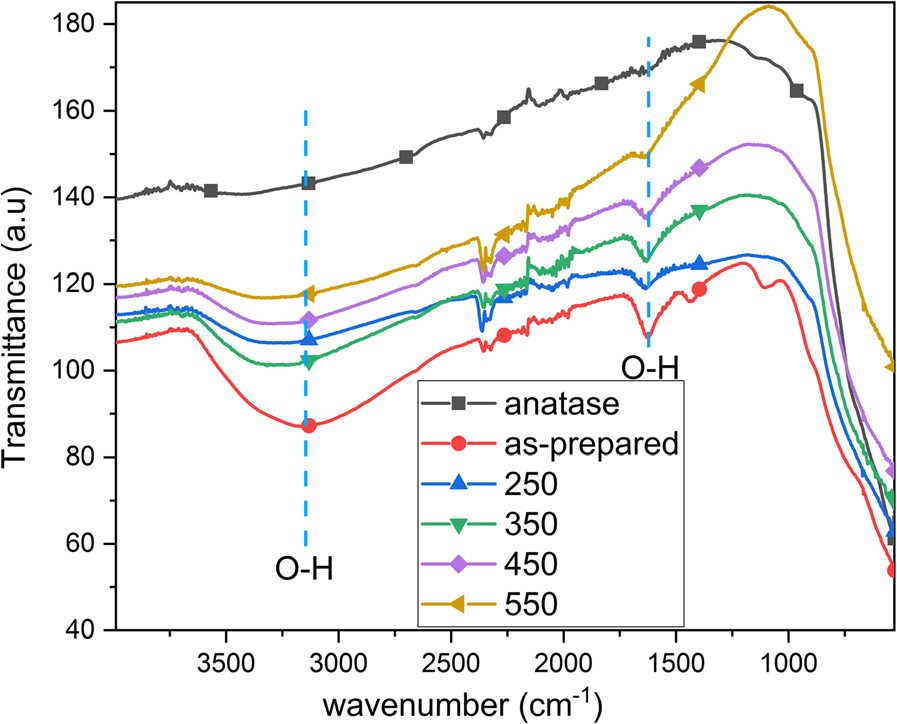
FTIR of as-prepared TNTs, annealed TNTs (250, 350, 450, and 550 °C) and reference anatase powder

The FTIR of the nanotubes and the reference anatase powder is shown in Fig. 5. The prominent O–H bending (1620–1640 cm^−1^) and stretching vibrations (3000–3500 cm^−1^) are observed for the as-prepared TNTs, similar to previous observations [11]. The O–H vibrations are also observed for the annealed TNTs, the intensity of which decreases with annealing temperature. No hydroxyl vibrations are observed for the reference anatase powder. The decrease in hydroxyl groups with annealing is similar to previous results [3, 11]. It should be noticed that the vibrations from 2000 to 2500 cm^− 1^ are due to an artifact from the equipment.

**Fig. 6:**
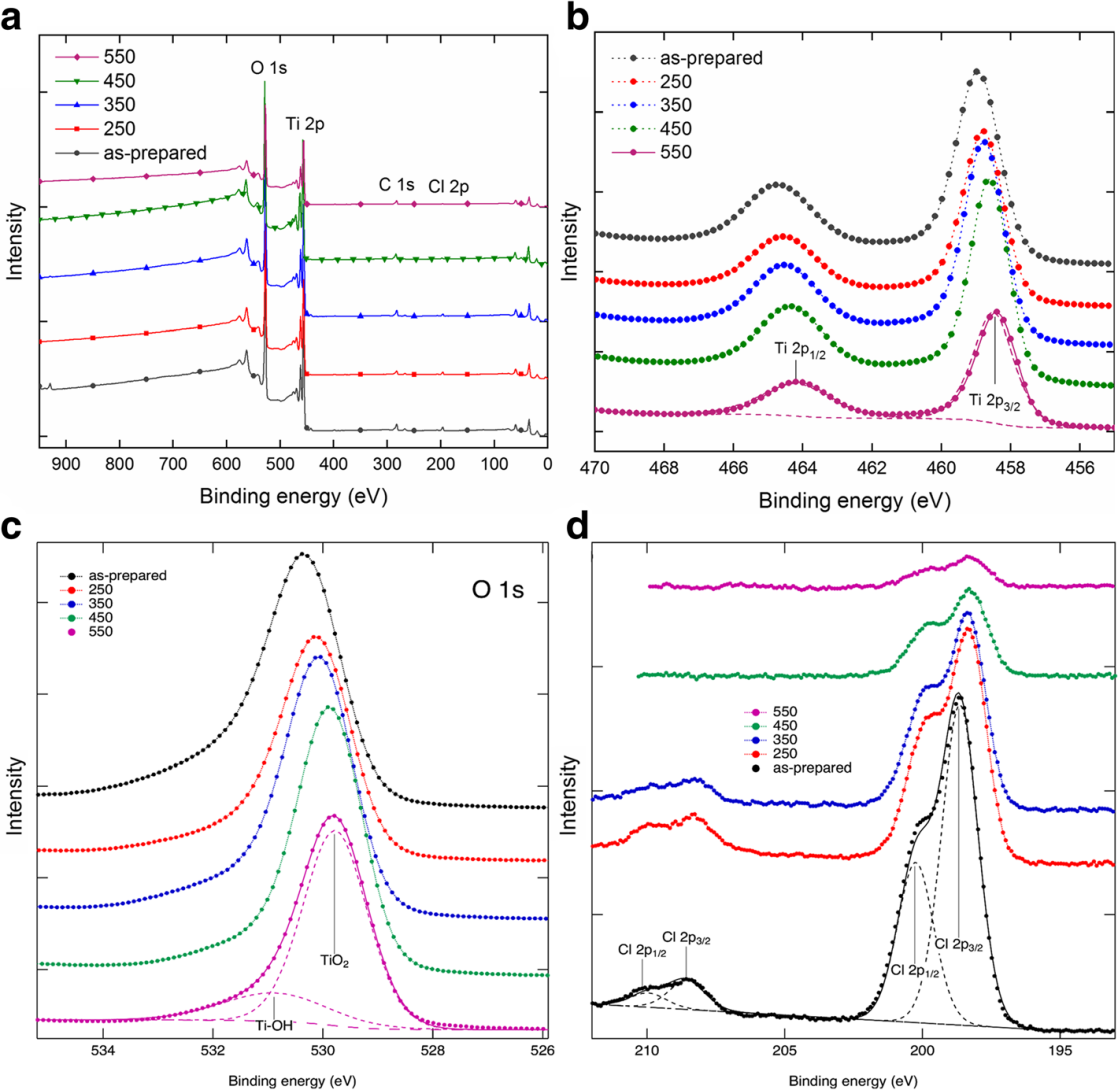
XPS spectra of TNT **a** survey spectra and high resolution scans of **b** Ti2p, **c** O1s, and **d** Cl2p

Figure 6 shows the XPS spectra obtained from the TNT surface. The survey spectra (Fig. 6a) were used to calculate the atomic percentages of the elements at the surface region shown in Table 1. The relative amount C is not shown in Table 1. Ti2p spectra shown in Fig. 6b show the characteristic peaks at 458.9 eV (Ti2p_3/2_) and 464.6 eV (Ti2p_1/2_) from the TNTs corresponding to TiO_2_ [31, 38–40]. The fitting was obtained using 40% Gaussian peaks. These peaks are found for the TNTs, but the binding energy on the 2p_3/2_ peak shifts gradually 0.4 eV downwards when the annealing temperature increases. This is in agreement with the phase change from anatase to rutile since the latter has a slightly lower Ti2p binding energy [41]. Figure 6c shows the O1s spectra of all the samples with the deconvolution made to the 550 °C annealed sample exhibiting Gaussian/Lorentzian (GL) components at the binding energy of 529.8 eV associated with O–Ti bonding [31, 38, 39, 42] and at 530.9 eV which is related to the presence of hydroxyl group with Ti (OH–Ti) [39, 42]. XPS shows a strong presence of chlorine on the as-prepared TNT surface. The spectrum in Fig. 6d has two Cl2p doublets, one at 198.6 eV (Cl2p_3/2_) and another at 200.1 eV (Cl2p_1/2_) [43–45]. These peaks are also present in all other samples as shown in Fig. 6d. Chlorine is a residue from the electrolyte, and its amount decreases upon annealing. Yang et al. [38] also reported the decrease in atomic concentration of fluoride ions with annealing for TNT arrays prepared in a fluoride electrolyte. As-prepared TNT, TNT 250, and TNT 350 have a peak at a binding energy of 208.5 eV (Cl2p_3/2_), related to the ClO_4_^−^ ionic bonding on the nanotube surface [46]. The high binding energy peaks disappear for samples annealed at higher temperatures, as in the case of TNT 450 and TNT 550 (Fig. 6d). Table 1 shows the atomic percentage of elements of TNT surfaces and reduction of Cl^−^ from 2.3 to 0.3% upon annealing. The ratio of Ti/O remains the same, contrary to a previous report [38].

**Table 1:** Quantification of elements based on XPS analysis

Sample	Ti (atomic %)	O (atomic %)	Cl (atomic %)
As-prepared	26	62	2.3
TNT 250	28	63	2.1
TNT 350	27	62	1.5
TNT 450	28	63	0.5
TNT 550	27	62	0.3

**Fig. 7:**
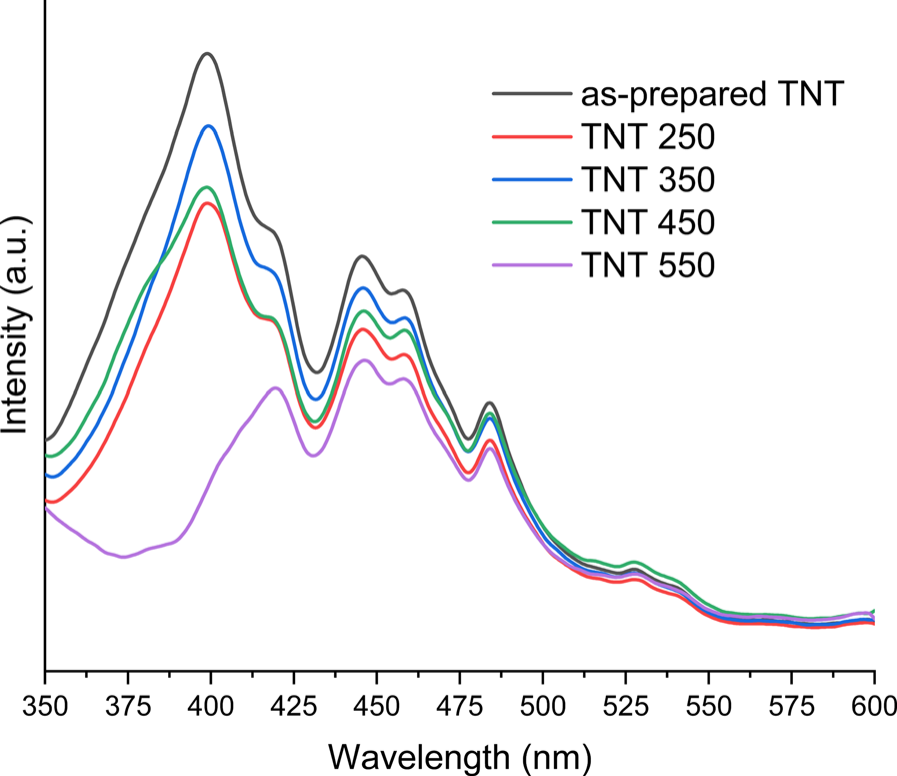
The photoluminescence emission spectra of as-prepared and annealed TNTs

PL is widely used to probe the photogenerated electrons in photocatalysts. Figure 7 shows the PL spectra of the TNT powder samples. All TNT samples, except TNT 550, have an emission band centered at 399 nm (3.11 eV). However, all samples share the emission band maximum at ca 419 nm (2.96 eV). These two emission bands support the bandgap values obtained from diffuse reflectance spectra as shown in Fig. 4. The shift in bandgap is also supported by the crystal structure change taking place in the TNT samples, as TNT 550 has a mixed anatase/rutile structure, and all other TNT samples have crystal structures of either anatase or anatase/brookite. Antony et al. found a similar red shift in the bandgap for their mixed anatase/rutile TNTs [34]. The PL intensities of the band at ca 419 nm could be put in the decreasing order of as-prepared TNT > TNT 350 > TNT 250 ≈ TNT 450 > TNT 550. The as-prepared TNTs show a higher recombination compared to the other TNT samples, probably due to more defects and lower crystallinity of the nanotubes. The lower PL intensity of TNT 250 and TNT 450 compared to TNT 350 is attributed to the mixed anatase/brookite structure, favoring electron transfer from anatase to brookite [47]. The intensity of TNT 550 also suggests increased charge separation and a longer lifetime of the electron-hole pairs as compared to the other TNT samples. Antony et al. [34] also reported the lowest PL intensities for rutile/anatase TNTs. However, photoinduced charge carrier lifetime is highly dependent on the rutile to anatase ratio [48]. The emission bands between 468 and 700 nm are attributed to the surface defects of TiO_2_ [17]. The typical defects in the as-prepared TNTs are Ti^3+^ sites, oxygen vacancies, and the partial coordination of Ti^4+^ [49, 50]. The partial coordination of Ti^4+^ can arise from anionic impurities (Cl^−^) originating from the preparation process [51]. The results obtained from XPS (Fig. 6) show chlorine contamination in all TNT samples; however, the concentration of the impurity decreases upon annealing. These impurities also contribute to the crystal defects which promote recombination [51]. The emission band maxima around 445 nm (2.79 eV) and 484 nm (2.56 eV) can be attributed to the surface states (O–Ti–OH) on the distorted octahedral TiO_6_ [34]. The emission bands around 539 nm (2.30 eV) [17] and 527 nm (2.36 eV) [52] are associated with oxygen vacancies in the titania structure. The emission band around 517 nm (2.40 eV) is associated with the Ti^4+^ ions near the oxygen vacancies [53].

**Fig. 8:**
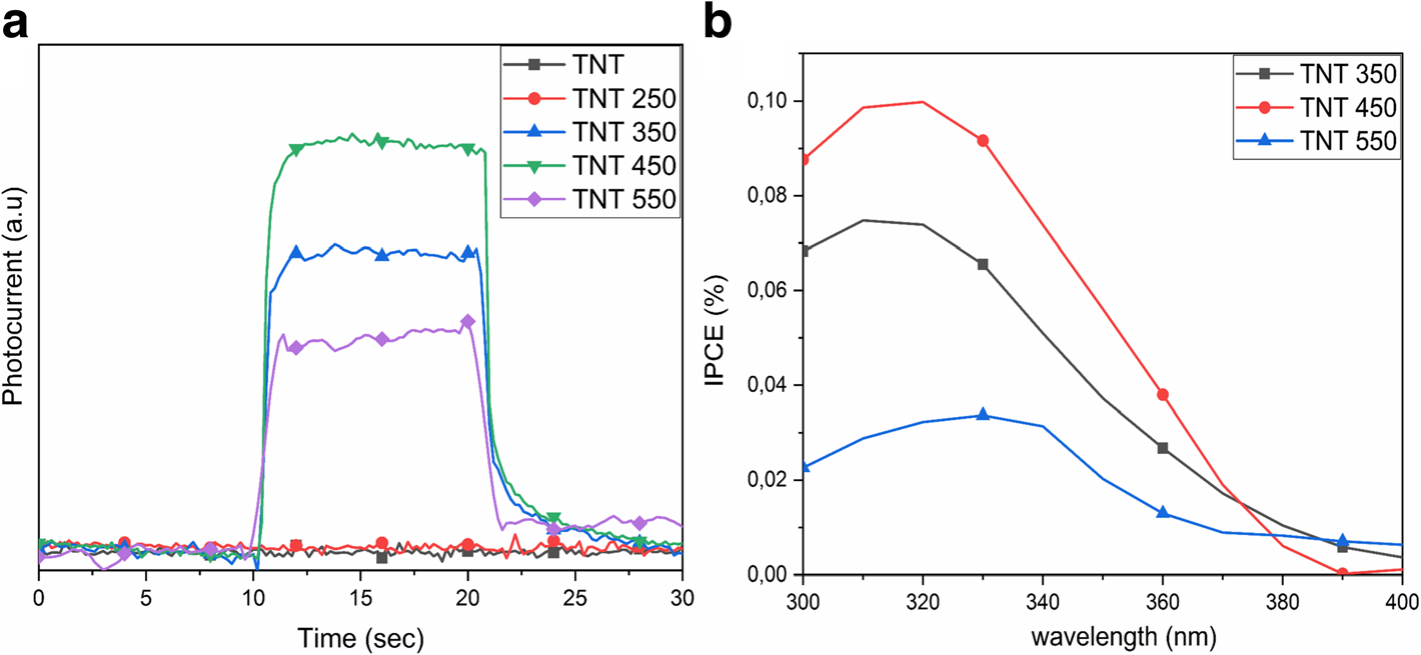
**a** The photocurrent obtained at the irradiation wavelength of 320 nm. **b** The IPCE values of the TNT 350, 450, and 550

Figure 8b shows the incident photon to current efficiency (IPCE) characterization of the TNT electrodes at irradiation wavelengths of 300–400 nm. The IPCE values are calculated from the following equation

2 $$ \mathrm{IPCE}\%=\frac{1240\times {J}_{\mathrm{ph}}}{\lambda \times {I}_{\mathrm{light}}} $$

where *J*_ph_ is the measured photocurrent density (mA cm^−2^), *λ* is the incident irradiated wavelength and *I*_light_ is the intensity of the light source (mW cm^−2^) at a specific wavelength (nm). The photocurrent is obtained for TNT 350, TNT 450, and TNT 550 upon irradiation. The highest photocurrent and IPCE values are obtained for the TNT 450 in case of mixed anatase/brookite structure. Brookite has a 0.14 eV more negative conduction band than anatase, which contributes to the electron transfer from brookite to anatase [47, 54]. The IPCE value of TNT 450 (anatase/brookite) is 1.37 times higher than TNT 350 (anatase) and 3.95 times higher than TNT 550 (anatase/rutile). TNT 550 (anatase/rutile) has the smaller photocurrent as compared to TNT 350 (anatase), which agrees well with previous findings [49, 55]. Previous studies show that a high rutile/anatase phase content decreases the photocurrent efficiency [56] and photocatalytic activity [57]. This agrees well with our findings as TNT 550 consists of clearly more rutile than anatase. However, no photocurrent could be obtained for the as-prepared and TNT 250 samples. The photocurrent is limited by trap-states present in the sample [49–51]. Previous studies have reported the appearance of photocurrent in TNTs annealed at 450 °C [2, 55, 58] or only a small photocurrent from the as-prepared TNT arrays [55]. However, the as-prepared TNT arrays were amorphous in those cases. Herein, a photocurrent is obtained for the TNT samples annealed at temperatures at or above 350 °C. The photocurrent efficiency is generally lower for RBA TNTs as compared to well-aligned TNT arrays, due to the inhomogeneous distribution of nanotube bundles [59]. The difference may result from the electrode preparation, possibly leaving ethanol residues in the TNTs or arise from the use of Nafion to adhere the powders to the FTO glass. The TNT electrodes were used for electrochemical impedance spectroscopy (EIS), and the results and analysis are found in the Additional file 1: Figure S3.

### Photocatalytic decolorization of TNTs

**Fig. 9:**
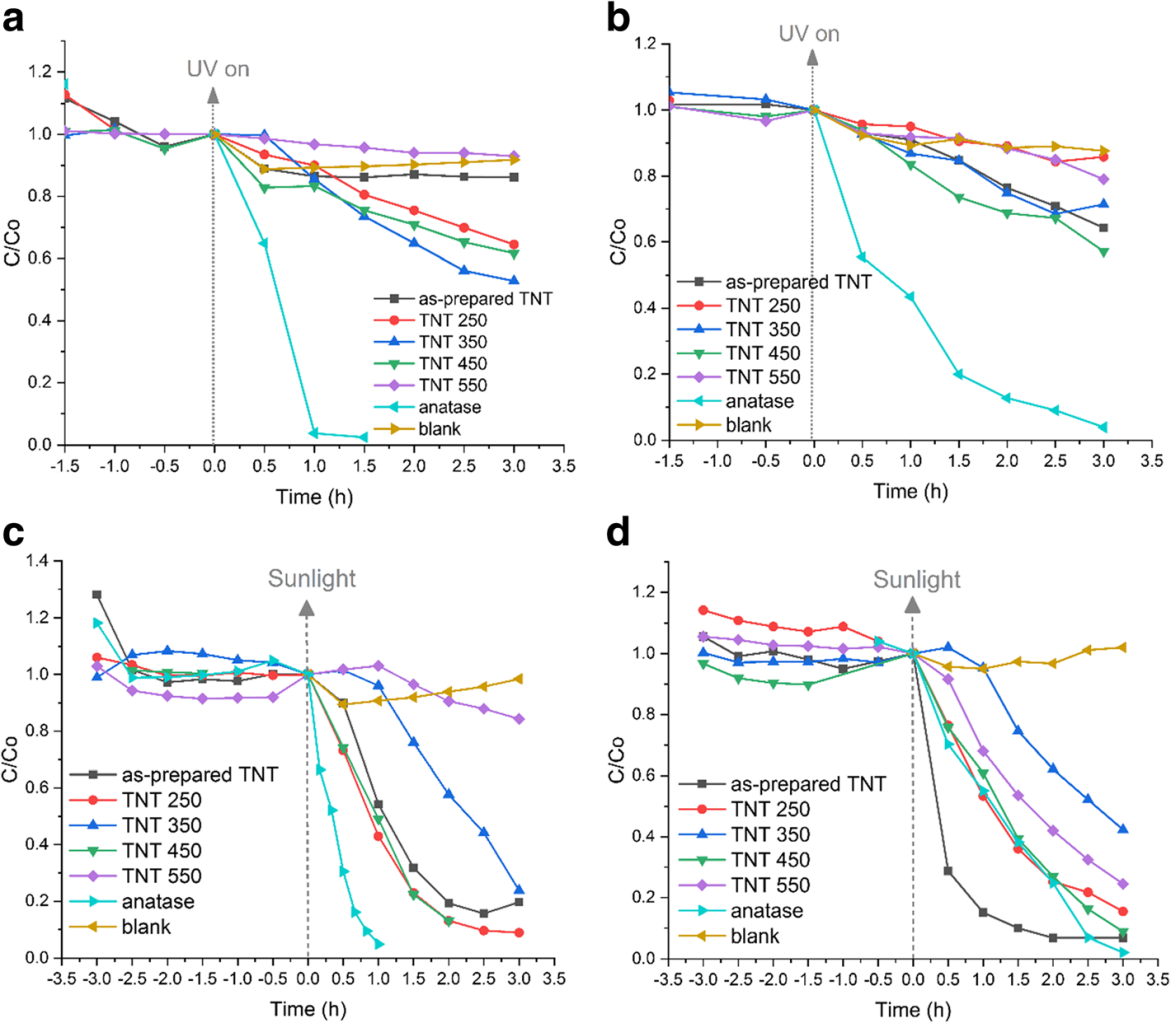
Photocatalytic decolorization of **a** MO and **b** RhB under UV light irradiation and **c** MO, and **d** RhB under sunlight irradiation for 3 h using the TNT powders

The adsorption and photocatalytic performance of the TNT powders was evaluated by photocatalytic decolorization of the anionic MO and cationic RhB dyes in aqueous solution under UV light and natural sunlight irradiation. The basic mechanism of photocatalytic degradation involves photogeneration of the electron-hole pair under irradiation. If the irradiation wavelength is greater than the bandgap of the material, the electrons are promoted from the valence band to the conduction band generating an electron-hole pair [26]. The photogenerated electron-hole pair reacts with water to form reactive hydroxyl (OH·) and superoxide radicals (O·^−^), which interact with the organic compound to decompose them to CO_2_ and water [25]. The schematic representation of the degradation mechanism for a single phase is proposed in Additional file 1: Figure S4. The mixed crystal structure of anatase/brookite (TNT 450) and anatase/rutile (TNT 550) with a phase-junction allows the mobility of electrons to flow from brookite to anatase [54] and from rutile to anatase [57]. This allows low electron-hole recombination upon excitation, and a schematic illustration is provided in Additional file 1: Figure S5. The adsorption capacity of the samples was examined prior to irradiation by keeping the samples under dark conditions until adsorption-desorption equilibrium was reached, i.e., within 3 h. The adsorption is shown in Fig. 9 as the time before irradiation begun. The largest adsorption of ca 28% was found for MO on the as-prepared TNT powder. All other samples adsorbed 15% or less of dye for both MO and RhB (Fig. 9).

Control experiments (blank tests) were performed for both dyes without a catalyst under UV light and sunlight irradiation. They showed a slight decrease in dye concentration under UV light: RhB concentration decreased by 14% and MO by 8%. The blank tests under sunlight displayed a 2% variation in concentration, which is within the margin of error of the measurement.

Figure 9a–b displays the decolorization of MO and RhB under UV light irradiation. The anatase reference powder decolorized both dye solutions within 3 h, whereas the best TNT powder for MO under UV light was TNT 350 with 47% of MO decolorized. TNT 250 and TNT 450 performed quite similarly with 36 and 38% of MO decolorized, respectively. For RhB, TNT 450 performed most effectively of the TNT powders with 43% of RhB decolorized within 3 h, while the as-prepared TNT powder was next with 36% of RhB decolorized.

The decolorization of MO and RhB under natural sunlight using RBA TNT catalysts are presented in Fig. 9c–d. It is notable that MO was completely decolorized by the as-prepared TNT, TNT 250, and the reference anatase powder within the detection limit of 1.0 mg L^−1^. TNT 450 decolorized MO up to 87% in 2 h. The fastest decolorization of RhB was observed using the as-prepared TNT, whereas the decolorization using TNT 250, TNT 450, and anatase reference powder was approximately equal. It is clear that the TNT powders perform significantly better under natural sunlight as compared to UV light.

**Fig. 10:**
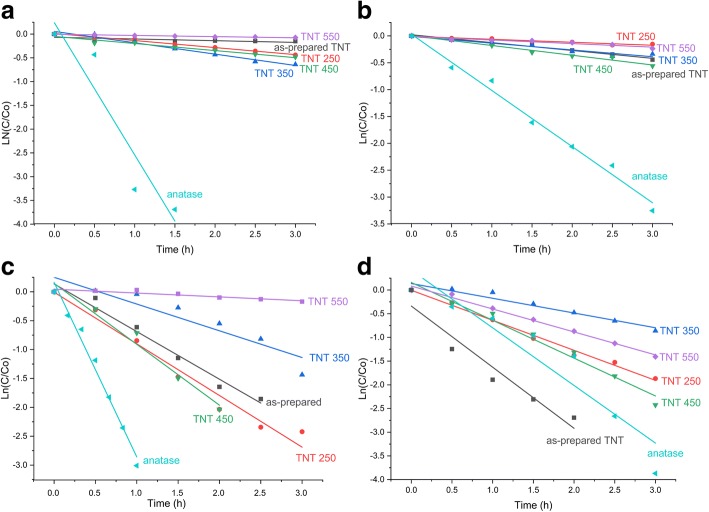
Pseudo-first-order plots of the decolorization of **a** MO and **b** RhB under UV light irradiation, and **c** MO and **d** RhB under natural sunlight irradiation

The data were fitted to the pseudo-first-order kinetic equation, which is expressed as ln(C/C_0_) = −*κ*_1_*t*, where *κ*_1_ is the first-order rate constant. By plotting ln(C/C_0_) against time (*t*), a straight line of which the slope equals the rate constant is obtained. All the determined rate constants and correlation factors (*R*^2^) are given in Table 2, and the fitting is shown in Fig. 10. The highest rate constants are found for the anatase reference powder under UV light, i.e., 2.78 h^−1^ for MO and 1.05 h^−1^ for RhB. For the TNT powders, the highest *κ*_1_ is found for TNT 350 (0.24 h^−1^) in MO and for TNT 450 (0.18 h^−1^) in RhB under UV light. The anatase reference powder displayed the highest rate constant at 3.02 h^−1^ in MO under sunlight, while TNT 450 yielded a *κ*_1_ of 1.05 h^−1^ under the same conditions. However, the as-prepared TNT in RhB performed better (rate constant of 1.29 h^−1^; *R*^2^ = 0.93) than the reference anatase powder (rate constant of 1.22 h^−1^; *R*^2^ = 0.89). The high photocatalytic activity of reference anatase powder in sunlight is ascribed to the UV light in the natural sunlight spectrum since the results are analogous under both UV and sunlight. The as-prepared TNT and TNT 450 have the two highest decolorization rates of the TNT powders for RhB; TNT 450 has the highest rate under UV light although the difference between the as-prepared TNT (0.15 h^−1^) and TNT 450 (0.18 h^−1^) is negligible. The as-prepared TNTs are the most efficient catalyst for RhB under sunlight.

**Table 2:** Specific surface area (SSA), bandgap, phase composition, and results from the photocatalytic tests using MO and RhB as model pollutants under UV and natural sunlight using TNT powders and reference TiO_2_ powder

				Methyl orange, MO								Rhodamine B, RhB							
				UV				Sunlight				UV				Sunlight			
Cat	Ph	SSA	Bandgap	*R*_eff_	*C*_(end)_	Reaction kinetics		*R*_eff_	*C*_(end)_	Reaction kinetics		*R*_eff_	*C*_(end)_	Reaction kinetics		*R*_eff_	*C*_(end)_	Reaction kinetics	
		m^2^ g^−1^	eV	%	mg L^−1^	*κ*_1_ h^−1^	*R* ^2^	%	mg L^−1^	*κ*_1_ h^−1^	*R* ^2^	%	mg L^−1^	*κ*_1_ h^−1^	*R* ^2^	%	mg L^−1^	*κ*_1_ h^−1^	*R* ^2^
TNT	A	179.2	3.04	13.9	6.6	0.04	0.50	80.3	1.1	0.82	0.97	35.7	5.5	0.15	0.98	93.2	0.7	1.29	0.93
250	A + B	123.3	3.03	35.6	6.7	0.15	0.99	91.1	1.0	0.89	0.97	14.2	8.4	0.06	0.94	84.6	1.4	0.63	0.99
350	A	86.5	3.07	47.2	5.6	0.24	0.98	76.2	2.6	0.46	0.87	28.6	6.2	0.13	0.94	57.7	4.3	0.31	0.94
450	A + B	70.4	3.14	38.3	7.4	0.15	0.96	87.0^a^	1.7	1.05	0.97	42.9	5.6	0.18	0.98	91.1	1.0	0.80	0.98
550	A + R	35.4	2.88	7.1	9.6	0.03	0.97	15.7	9.7	0.07	0.85	21.0	7.5	0.07	0.92	75.5	2.3	0.48	0.99
TiO_2_	A	10.2	3.18	97.5	0.3^*^	2.78	0.89	95.1^b^	0.4^*^	3.02	0.98	96.1	0.2	1.05	0.99	97.9	0.2	1.22	0.89

*Cat* catalyst, *Ph* phase, *R*_*eff*_ removal efficiency, *A* anatase, *R* rutile, *B* brookite, *TiO*_*2*_ reference powder

^a^After 2 h

^b^After 1 h

^*^Below the detection limit

**Fig. 11:**
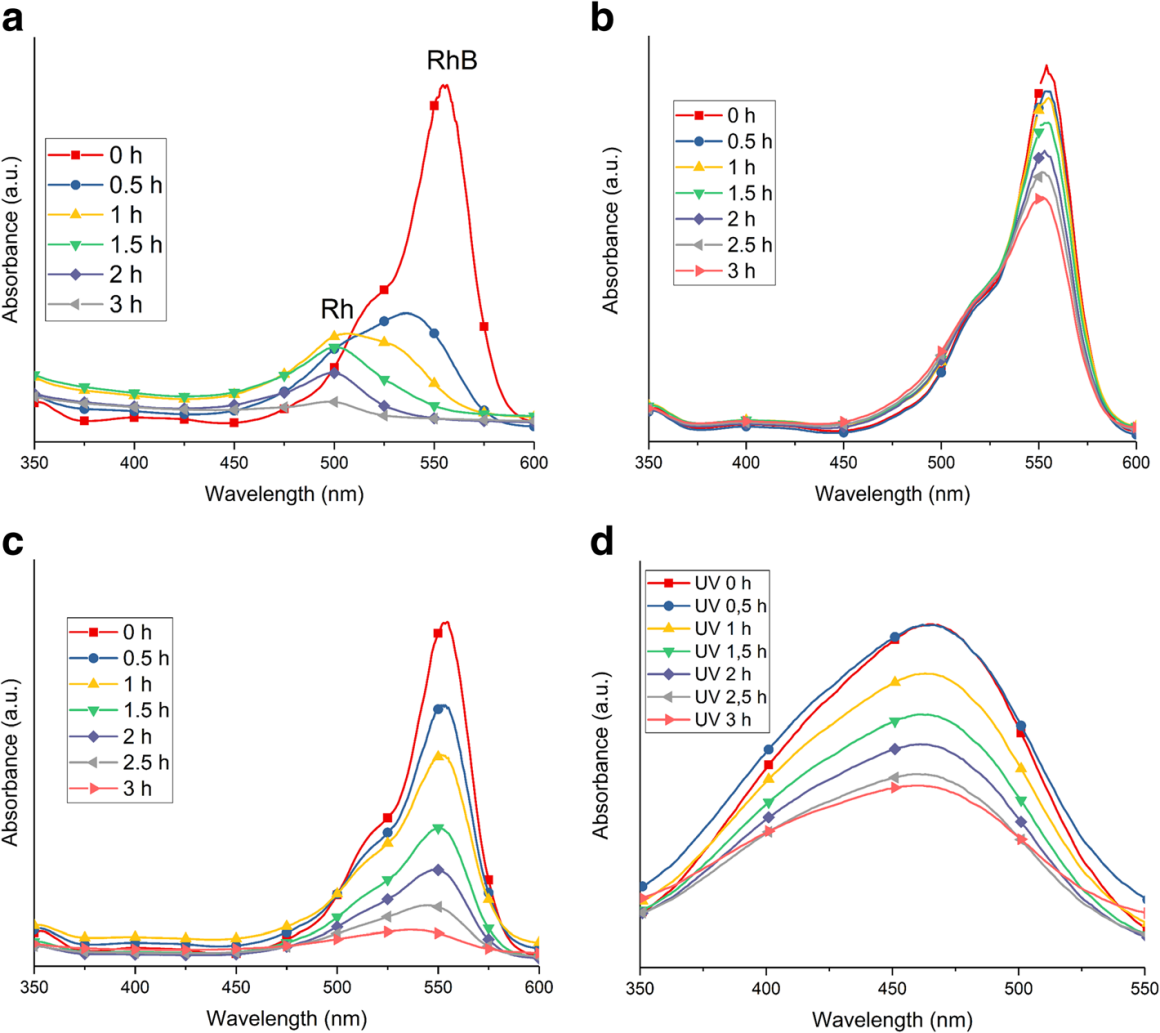
UV-Vis absorbance spectra of the decolorization of RhB using **a** the as-prepared TNT powder under sunlight, **b** the as-prepared TNT powder under UV light, **c** the TNT 450 powder under sunlight and the decolorization of MO by **d** TNT 350

The main differences between the as-prepared TNT and TNT 450 powders are in their structure, specific surface area, the atomic concentration of chloride impurity, and electron-hole lifetime. The as-prepared TNTs are tubular consisting of anatase phase with the high specific surface area of 179 m^2^ g^−1^, while the TNT 450 are rods consisting of anatase and brookite phases with a specific surface area of 70 m^2^ g^−1^. The PL and IPCE results suggest better conductivity of TNT 450 as compared to as-prepared TNTs. The absorption spectra in Fig. 11 show that there is a clear difference in the photocatalytic mechanism between the as-prepared TNTs under sunlight (Fig. 11a) and UV light irradiation (Fig. 11b). Also, the degradation processes of RhB for TNT 450 (Fig. 11c) are clearly different from as-prepared TNTs. No peak shift (*λ*_max_ = 554 nm) is observed under UV light, only a decrease in absorbance for the RhB peak. Two types of changes are observed under sunlight: First, the absorbance for RhB decreases, and second, the peak shifts to 498 nm. The peak at 498 nm is identified as Rhodamine, which is an N-de-ethylation product of RhB [60, 61]. N-de-ethylation only takes place under visible light, in this case, the visible light spectrum from the solar light irradiation, due to the excited RhB molecules [62]. The adsorbed and excited RhB molecules then transfer the excited electron to the conduction band of the TNT powder. This step also confirms the degradation of RhB to Rhodamine by N-de-ethylation and further to smaller components [62–64]. No other catalyst displayed Rhodamine as an intermediate product during decolorization of RhB. The N-de-ethylation reaction is dependent on the formation of OOH^·^ and OH^·^, which is suggested to be more prominent in the reaction of the as-prepared TNTs with RhB. The as-prepared TNT carries more –OH functional groups on its surface (Fig. 5) and results in excellent dispersion of the as-prepared powder in the aqueous solution [62]. The N-de-ethylation only takes place on as-prepared TNTs in our case, similar to the study by Guo et al. [65], where they only observed N-de-ethylation on their titanate nanotubes. The justification for this phenomenon may be that nanorods have a lower adsorption capacity associated with the reduction of the specific surface area upon annealing [65]. The decolorization reaction of RhB using TiO_2_ materials under UV light is different and requires the formation of RhB^+·^ and the presence of air [65]. The mechanism has been widely studied elsewhere [65].

The photocatalytic decolorization of MO using the TNT powders presents a different trend between the samples of which one has an increased decolorization effect. Under UV light, TNT 350 presented the most prominent kinetic rate of 0.24 h^−1^, whereas for sunlight, the most prominent rate was found for TNT 450 with 1.05 h^−1^; however, the TiO_2_ anatase reference powder was superior in both cases. Despite the comparatively small specific surface area, TNT 450 with the mixed crystal structure (anatase/brookite) exhibited the best photocatalytic efficiency of the prepared TNTs. This is attributed to the improved electron-hole separation, apparent from high photocurrent obtained from TNT 450 in Fig. 8. This result is consistent with published reports [37, 47]. The adsorption spectra from the MO show only the decrease in absorbance with irradiation time (Fig. 11d). No blueshift is observed for the decolorization of MO for any TNT catalyst, suggesting the damage to the conjugated system made up of azo groups and aryl rings of MO dye [65].

Titanate nanotubes with different morphology and crystal structure have been tested under simulated solar light and have reached almost complete degradation of MO and RhB [65]. RBA TNT powders as photocatalysts have been investigated under UV light using MO [20] and acid orange 7 (AO7) dye [21]. Liao et al. [20] reported a reaction rate of 0.082 min^−1^ (4.92 h^−1^) which is significantly higher than the rates obtained in this study under UV light. However, it is noteworthy that the post-treatment destroyed the tubular structure of the TNTs into particles (Liao et al. [20]). Hahn et al. [21] reported decolorization rates for doped TNT layers up to 0.469 h^−1^ (undoped TiO_2_ 0.385 h^−1^) using AO7 as a model dye under UV light irradiation. It is crucial to use a catalyst that can utilize the extended sunlight spectrum for efficient degradation of pollutants. RBA TNT powders prepared under different washing conditions and annealed at 500 °C, with a pure rutile phase have been reported as an efficient photocatalyst for RhB degradation under visible light illumination [33]. In the present report, the study of photocatalytic properties of TNT powders prepared by RBA is extended from UV to visible light range under sunlight irradiation, contrary to the general perception of titania only being active under UV light.

## Conclusions

The TNT powders were prepared by rapid breakdown anodization. By annealing the TNT powders at 250–550 °C, the bandgap narrowed from 3.04 eV down to 2.88 eV, and the tubes transformed from anatase phase to anatase/brookite and further to anatase/rutile phase mixtures. The tubes transformed to nanorods at 350 °C, reducing the specific surface area from 179 to 35 m^2^ g^−1^. The XPS results show the characteristic peaks of Ti2p and O1s and Cl2p in all TNTs; however, the atomic concentration of chlorine decreases upon annealing. The PL results for TNT 250 and TNT 450 suggest lower electron-hole recombination as compared to as-prepared TNT and TNT 350. The higher recombination in the as-prepared TNTs is attributed to the low crystallinity and the number of surface defects. The photocatalytic activity of the TNT powders was investigated by decolorization of MO and RhB dyes under UV and natural sunlight. The photocatalytic decolorization of both dyes improved under natural sunlight, contradicting the general perception of titania nanotubes being inefficient photocatalysts under visible light irradiation. The as-prepared TNTs, TNT 250, and TNT 450 performed the best of the TNT powder samples under natural sunlight using RhB and MO as model pollutants, whereas the as-prepared TNT powder outperformed the reference TiO_2_ anatase powder when using RhB as a model pollutant. This could be attributed to the higher specific surface area and different photocatalytic degradation mechanism of RhB on the as-prepared TNT. TNT 250 and TNT 450 displayed similar activity under sunlight irradiation, which is ascribed to the large surface area and reaction sites for TNT 250. The specific surface area is reduced for TNT 450, and the better photocatalytic activity is attributed to a favorable crystal structure and less electron-hole recombination. In addition, the highest IPCE values are obtained for TNT 450. These RBA TNTs may intensify the use of the natural sunlight spectrum for removal of organic contaminants from polluted waters.

## Additional file

Additional file 1: **Figure S1.** TEM micrographs of **a** TNT 350, **b** TNT 450, and **c** TNT 550. **Figure S2.** Raman spectra of TNT as-prepared, TNT 250, TNT 350, TNT 450, and TNT 550. The inset shows the brookite peaks from TNT 550. (DOCX 1134 kb)

## Abbreviations

DRS: Diffuse reflectance spectroscopy
EIS: Electrochemical impedance spectroscopy
FTIR: Fourier transform infrared spectroscopy
FTO: Fluorine-doped tin oxide
IPCE: Incident photon to current efficiency
MO: Methyl orange
PL: Photoluminescence
RBA: Rapid breakdown anodization
RhB: Rhodamine B
SEM: Scanning electron microscopy
TEM: Transmission electron microscopy
TNTs: Titania nanotubes
XPS: X-ray photoelectron spectroscopy
XRD: X-ray diffraction
