# Supplementary material for: Titania nanotubes prepared by rapid breakdown anodization for photocatalytic decolorization of organic dyes under UV and natural solar light
Source: Nanoscale Res Lett. 2018 Jun 14;13:179. doi: 10.1186/s11671-018-2591-5 (PMC5999600; doi:10.1186/s11671-018-2591-5)
Supplement: Supplementary file 1 — Figure S1. TEM micrographs of a TNT 350, b TNT 450, and c TNT 550. Figure S2. Raman spectra of TNT as-prepared, TNT 250, TNT 350, TNT 450, and TNT 550. The inset shows the brookite peaks from TNT 550. (DOCX 1134 kb) [file 11671_2018_2591_MOESM1_ESM.docx]

**Additional file 1**

**Titania nanotubes prepared by rapid breakdown anodization for photocatalytic decolorization of organic dyes under UV and natural solar light**

Saima Ali,^a^†* Henrika Granbohm^a^†, Jouko Lahtinen^b^ and Simo-Pekka Hannula^a^

^1^Department of Chemistry and Materials Science, Aalto University School of Chemical Engineering, P.O. Box 16100, FI-00076, Finland.

^2^Department of Applied Physics, School of Science, Aalto University, P.O. Box 15100, FI 00076 Aalto, Finland

^*^These two authors made equal contributions

^†^Corresponding author

Email: [saima.ali@aalto.fi](mailto:saima.ali@aalto.fi) and [henrika.granbohm@aalto.fi](mailto:henrika.granbohm@aalto.fi)

Results and discussion

**

Figure S1** TEM micrographs of **a** TNT 350 **b** TNT 450 and **c** TNT 550



**Figure S2** Raman spectra of TNT as-prepared, TNT 250, TNT 350, TNT 450 and TNT 550. The inset shows the brookite peaks from TNT 550

The electrochemical impedance spectra is depicted as a Nyquist plot for the as-prepared TNT, TNT 250, TNT 350 and TNT 450 in **Figure S3**. No clear semicircle to acquire the capacitance and charge transfer resistance is observed, which might be due to the Ru(NH_3_)_6_^2 +/3 +^ redox pair diffusing through the whole layer of TNT due to the porosity of the film and the redox reactions taking place straight at the FTO-glass surface. Therefore, no certain conclusions can be drawn concerning the charge transfer process in the TNT films on the electrodes nor for the TNT powders.


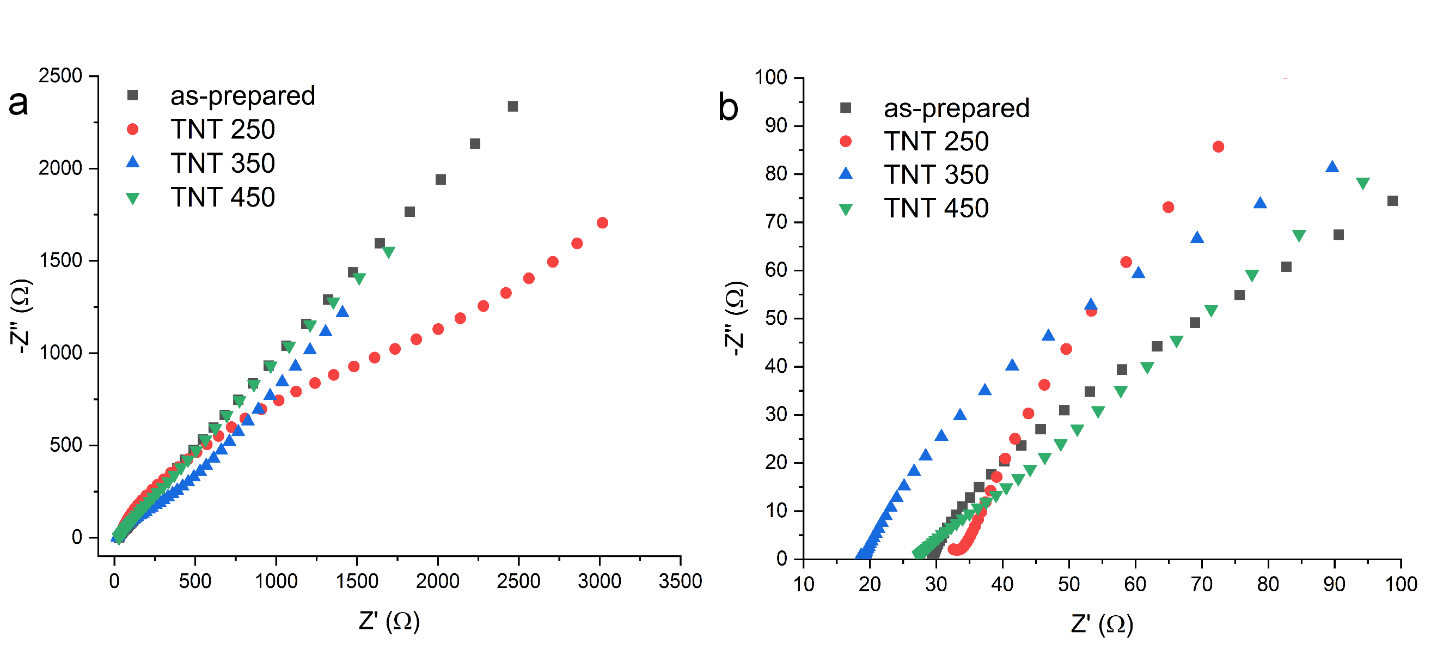


**Figure S3** **a** Electrochemical impedance spectroscopy (EIS) measurements of the as-prepared TNT, TNT 250, TNT 350 and TNT 450 and **b** partial magnification of the EIS plot at high frequencies


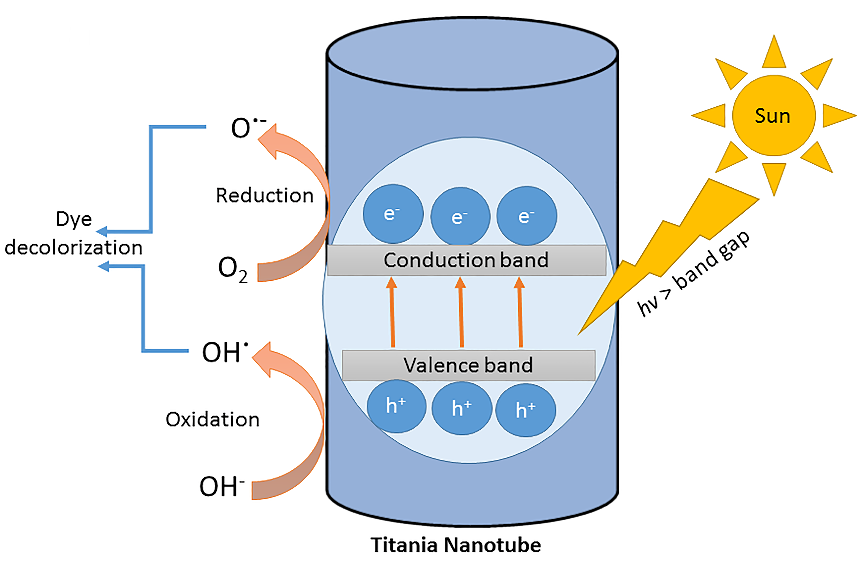


**Figure S4** The schematic representation of photocatalytic degradation mechanism with a single phase, such as anatase. h^+^ represents the holes and e^-^ shows the electrons. Adapted from [1]


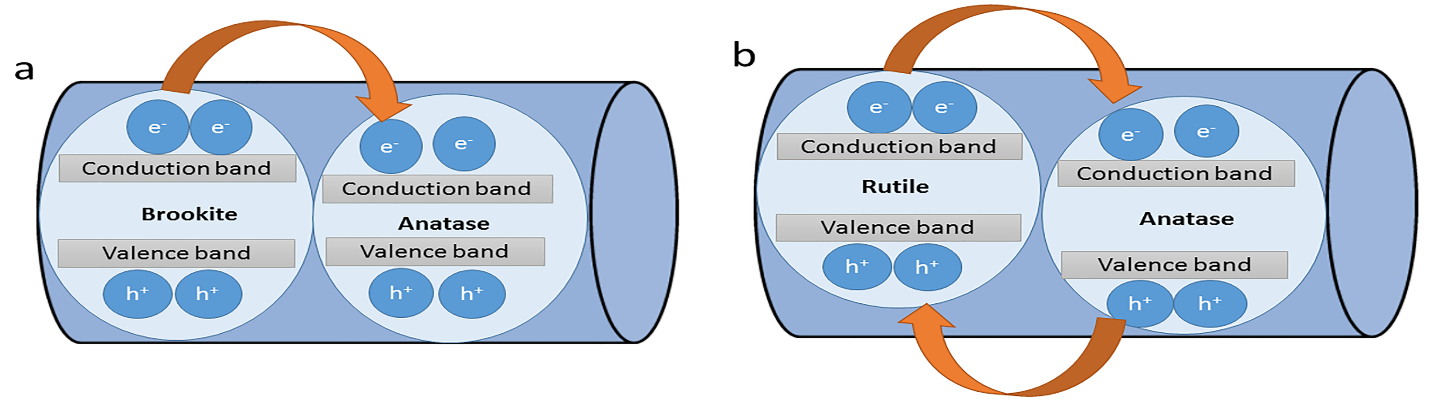


**Figure S5** A proposed schematic illustration of interfaces in **a** anatase/brookite and **b** anatase/rutile heterojunctions. h^+^ represents the holes, e^-^ shows the electrons and arrows indicates the movement of electrons and holes. Adapted from [2,3]

References

[1] M. Pelaez, N.T. Nolan, S.C. Pillai, M.K. Seery, P. Falaras, A.G. Kontos, P.S.M. Dunlop, J.W.J. Hamilton, J.A. Byrne, K. O’Shea, M.H. Entezari, D.D. Dionysiou, A review on the visible light active titanium dioxide photocatalysts for environmental applications, Appl. Catal. B Environ. 125 (2012) 331–349. doi:10.1016/j.apcatb.2012.05.036.

[2] Q. Tay, X. Liu, Y. Tang, Z. Jiang, T.C. Sum, Z. Chen, Enhanced Photocatalytic Hydrogen Production with Synergistic Two-Phase Anatase/Brookite TiO2 Nanostructures, J. Phys. Chem. C. 117 (2013) 14973–14982. doi:10.1021/jp4040979.

[3] W.-K. Wang, J.-J. Chen, X. Zhang, Y.-X. Huang, W.-W. Li, H.-Q. Yu, Self-induced synthesis of phase-junction TiO_2_ with a tailored rutile to anatase ratio below phase transition temperature, Sci. Rep. 6 (2016) 20491. doi:10.1038/srep20491.
